# Discovery and characterization of substrate- and product-selective nylon hydrolases

**DOI:** 10.1063/4.0001115

**Published:** 2025-10-27

**Authors:** Nikolas Capra, Liangyu Qian, Célestin Bourgery, John F Cahill, Alexis Williams, Dana Carper, Jerry Parks, Isaiah T Dishner, Jeffrey C Foster, Delyana Vasileva, Serena Chen, Joshua Michener, Flora Meilleur

**Affiliations:** 1 Oak Ridge National Laboratory, Oak Ridge, TN, USA; 2 North Carolina State University, Raleigh, NC, USA

## Abstract

Nylons are common industrial polyamides with few effective recycling options. As an alternative to mechanical or chemical recycling, enzymes could provide a selective and energy-efficient route to deconstruct nylons from mixed waste. Several nylon hydrolases have been identified (NylA, NylB, NylC), but the characterized enzymes demonstrate similar activity and substrate promiscuity. We synthesized and characterized a panel of 95 diverse enzymes from the Ntn-hydrolase superfamily with 30-50% pairwise amino acid identity. Several newly-identified hydrolases had promiscuous nylon hydrolase activity, in many cases comparable to that of best-characterized nylon hydrolase NylC from Flavobacterium sp. These enzymes showed significant substrate selectivity up to about 20-fold selective towards PA66 or 4-fold for PA6, which open new opportunities for selective deconstruction of polyamide waste. Furthermore, in an effort to upcycle these hydrolysis products, we identified a set of enzymes capable of catalyzing the synthesis of asymmetrical triads and tetrads from nylon-relevant monomers. Recently, we determined crystal structures and oligomerization states of a set of nylon hydrolases to elucidate structural determinants of the enzyme functional form. The oligomer-distribution studies suggested a non-trivial, concentration-dependent oligomerization. The crystal structures revealed two different PEG molecules from the crystallization buffer bound in different portions of a tunnel-like cavity, allowing for the identification of putative binding sub- pockets in the vicinity of the active site. This structural information can provide insights into the evolution of nylon hydrolase activity and opportunities for the engineering of improved hydrolases. Moreover, with respect to the oligoamide-synthesizing enzymes, we identified a set of homologs of the acyl-AMP ligase SfaB and engineered key residues by integrating structural analysis and machine learning methods, resulting in improved enzymatic activity for oligomer synthesis toward the products of catalysis by nylon hydrolases.

